# Management guidelines for amelogenesis imperfecta: a case report and review of the literature

**DOI:** 10.1186/s13256-020-02586-4

**Published:** 2021-02-09

**Authors:** M. Roma, Puneet Hegde, M. Durga Nandhini, Shreya Hegde

**Affiliations:** 1 Department of Conservative Dentistry and Endodontics, Manipal College of Dental Sciences, Mangalore, Manipal Academy of Higher Education, Manipal, Karnataka India; 2 Department of Prosthodontics and Crown and Bridge, Manipal College of Dental Sciences, Mangalore, Manipal Academy of Higher Education, Manipal, Karnataka India; 3 Department of General Medicine, Government Kilpauk Medical College, Chennai, Tamil Nadu, India

**Keywords:** Amelogenesis imperfecta, Oral rehabilitation, Enamel disorder, Hypoplastic enamel

## Abstract

**Background:**

Rehabilitation of the entire dentition with amelogenesis imperfecta (AI) tends to pose a great challenge to the clinician. Most of the cases of amelogenesis imperfecta are reported to be associated with skeletal and dental deformities which results in severe sensitivity of the dental tissues.

**Case presentation:**

This clinical case report marks out the total restoration of the oral condition of a young Indian patient diagnosed with the hypoplastic type of amelogenesis imperfecta. Fixed metal ceramic prosthesis were planned to strengthen the masticatory activity, aesthetics, to banish the dental sensitivity and to build up the general persona of the patient. The patient was followed-up at 6 months, 1 year and 2 years intervals. Functional and esthetic impairment was not visible after the follow up period and the treatment outcome was successful. The entire treatment plan was intended to enhance the functional, esthetic and the masticatory component of the occlusal architecture.

**Conclusion:**

This case report details the presentation, characteristic radiographic findings, and management of a patient with an extremely rare condition of amelogenesis imperfecta.

## Introduction

Enamel is considered as the hardest and the highly mineralized tissue in the human architecture, with 85% of the volume enveloped by hydroxyapatite crystals [1, 2]. The physiological morphology is forthwith related to compositional microstructure, direction and orientation of enamel rods, and structural configuration of the mineral constituent of the tissue [3, 4]. In the course of organogeny, the metamorphosis of enamel takes place from cushy and pliant structure to hard mineralized form which completely lacks protein [5] The final structural architecture is mirror image of the various developmental and cellular activities that occur during morphogenesis [4] Any divergence from the normal organogenesis leads to amelogenesis imperfecta which can be manifested in the oral cavity.

Amelogenesis imperfecta is of genomic origin affecting the structural, physical and clinical appearance of the individual which can be demotivating patient’s self-esteem. Such cases occur in one in million and are unique. They need to be handled with extreme care, vigilance and precision keeping in mind the soft and pliable nature of enamel which chips away easily. Hence, this case report marks the treatment strategy of handling such rare cases in medical and dental fields with utmost care and interdisciplinary approach to strengthen the maxillofacial architecture.

## Case report

A 23-year-old Indian female patient reported to the Department of Conservative Dentistry and Endodontics with a chief complaint of yellowish discoloration and poor appearance of her teeth with the rehabilitation of the same. Family history and medical history was non-contributory. Patient was unmarried, well qualified medical student and there was no history of similar condition in the family. Her parents and siblings were fine and no other family member presented with similar dental findings. Her past dental history disclosed that she underwent root canal treatment of 36 due to caries involvement. Extra-oral examination disclosed normal mouth opening and no temporomandibular joint problems. The patient was healthy with no co-morbidities.

**Fig. 1.**
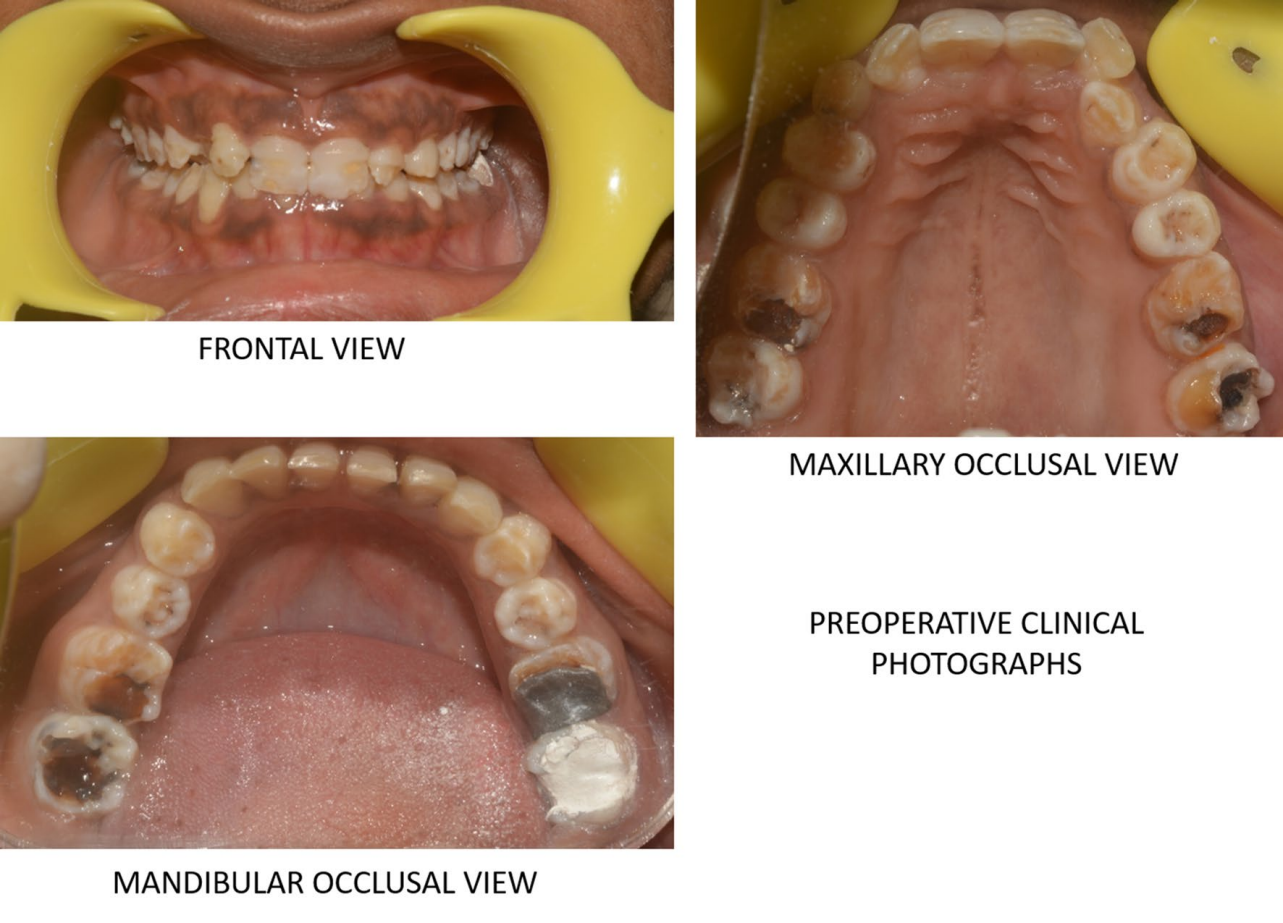
Preoperative clinical photographs showing teeth with well-defined pitted surfaces along with yellowish brown discoloration and normal healthy periodontium.

On intraoral examination, the enamel thickness was highly thinned down and partially flaked off from few surfaces of teeth. Teeth revealed well defined pitted surfaces along with yellowish brown discoloration. Surfaces of teeth were rough with minimal enamel present. The teeth also revealed short clinical crowns with multiple spacing between teeth. The occlusal plane was uneven worn-down posterior teeth. On frontal view, the patient presented with deep bite with less horizontal overlap. Dentin which was exposed were brown in color. The periodontal examination revealed normal healthy gingiva with no abnormalities (Fig 1).

**Fig. 2.**
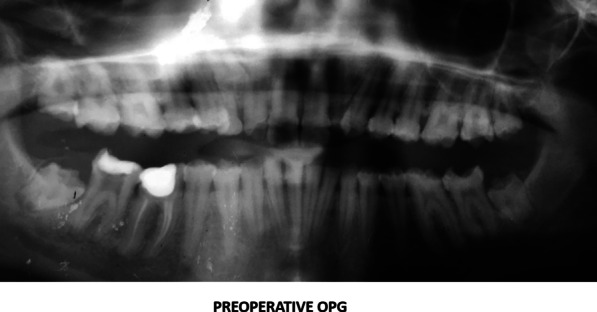
Preoperative orthopantomogram view showing root canal treated tooth with respect to 36 and the endodontic treatments to be performed on #37, 47,47,16,26

Radiographic examination included full mouth Orthopantomogram (OPG) and Intraoral periapical radiograph (IOPA) showed features of AI. (Fig 2) Occlusion was assessed after obtaining the diagnostic casts. Based on clinical and radiological examination, it was diagnosed as hypoplastic type of amelogenesis imperfecta. Based on the arrived diagnosis, a full mouth rehabilitation was planned. A well formulated treatment plan involving endodontic, and prosthetic department was charted out to furnish a functional occlusion with good esthetics and further prevent the tooth loss. The entire treatment plan was explained to the patient and informed consent was obtained. The initial phase of the treatment consisted of endodontic therapy. Based on OPG reports, it was decided that pulp space therapies should be performed on 37, 46, 47, 16, 17, 26 followed with retreatment with respect to 36 and fixed prosthesis was planned for the entire dentition.

Treatment protocol: Patient was asked to check her on diet to maintain oral hygiene. The mock presentation of full coverage prosthesis were executed on upper and lower casts and presented to the patient. She was contented with the anticipated outcome and prognosis of the overall treatment plan was satisfactory. The subsequent rehabilitation plan was formulated.

Definitive treatment plan:

1. Patient motivation for the treatment and maintenance of oral hygiene,
2. Endodontic treatments of #37, 47, 47, 16, 26,
3. Providing temporary bridges on all posterior teeth for reconstruction of the lost occlusal vertical dimension,
4. Trial of fixed partial dentures and,
5. Periodic follow-ups at 1 year and 2 year intervals followed by augmentation of oral hygiene measures.

*Endodontic treatment* was performed with respect to #37, 47, 47, 16, 26 under rubber dam and local anesthesia. 3% sodium hypochlorite (Vishal Dentocare Pvt Ltd, India) was used as irrigant in all the treated teeth. The working lengths for all teeth was obtained using hand K file (Dentsply Inc, Maillefer, Dentsply India), Root ZX II Apex locator (Morita, Irvine, CA), and finally confirmed using intraoral periapical radiograph. #16, 26 showed the presence of MB2. Cleaning and shaping of all the canals were performed with hand K files and Protaper rotary files (Dentsply Inc, Maillefer, Dentsply India) under copious irrigation with 3% sodium hypochlorite and enlarged upto F2 file size. Retreatment was performed with respect to #36. Three rounds of calcium hydroxide as intracanal medicament (CALCICURE, Voco) were placed for #36. All the access cavities were given temporary seal dressing (cotton pellet and intermediary restorative material, Cavit G, 3M ESPE GmbH, Neuss, Germany). All the obturation was completed using F2 protaper gutta percha cone (Dentsply, Maillefer, Dentsply India) with AH Plus sealer (Dentsply, Sirona, Dentsply India). The next stage of treatment was the restorative and prosthetic phase. Fixed partial dentures were planned.

*Restorative treatment* included the Post endodontic restoration of # 37, 46, 47, 16, 17, 26 and 36 with composite restoration (Filtek Z 350 XT, 3M ESPE, St, Paul, MN, USA). As per radiographic findings, the impacted teeth was retained as the patient was not willing for any surgical extrusion procedure. The deciduous 63 was included in the tooth preparation. All third molars (#18, 28, 38, 48) were not extracted as they had not erupted.

**Fig. 3.**
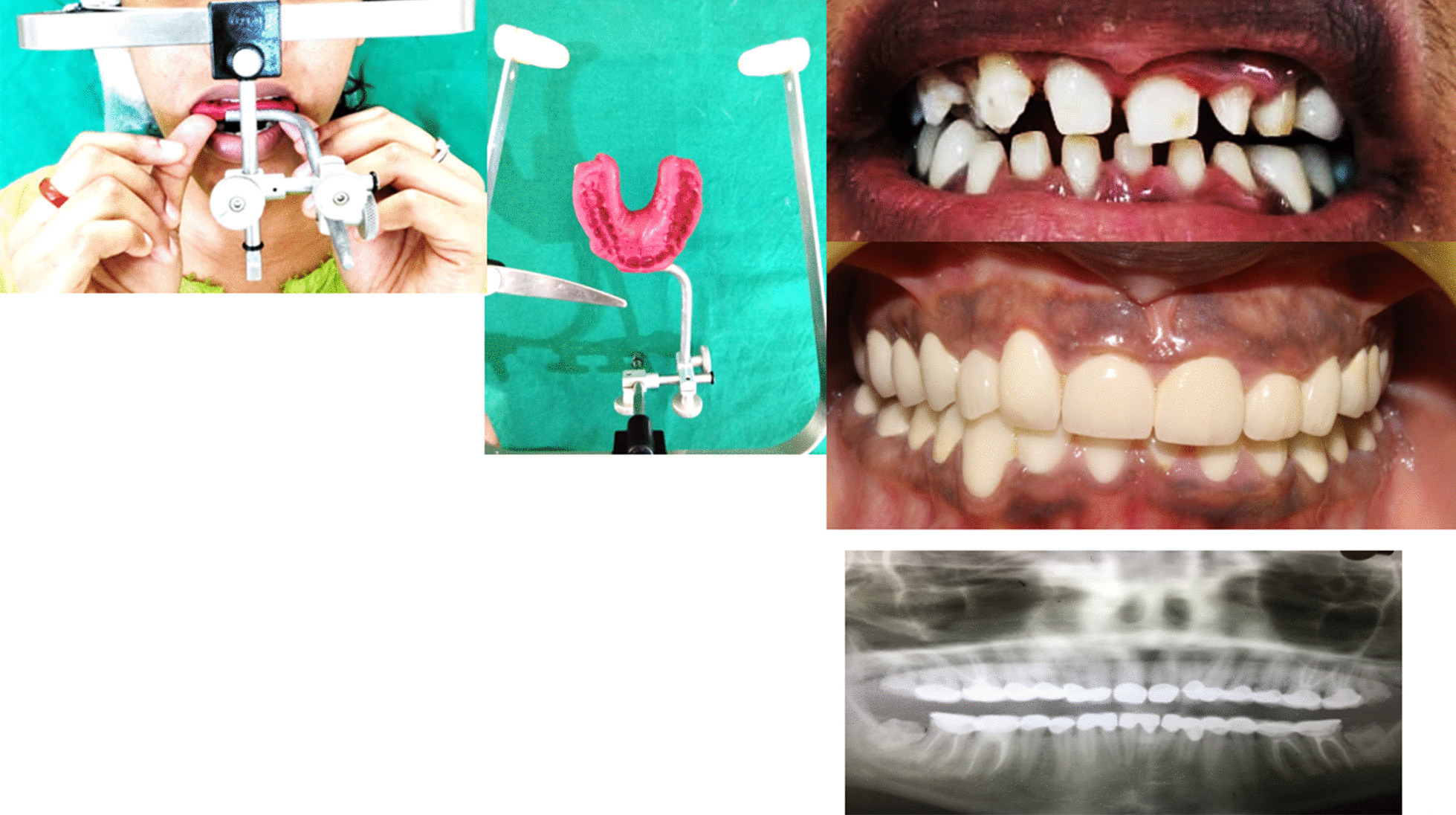
Clinical photographs showing facebow transfer, and the clinical procedures for prosthetic rehabilitation and following postoperative OPG view.

**Fig. 4.**
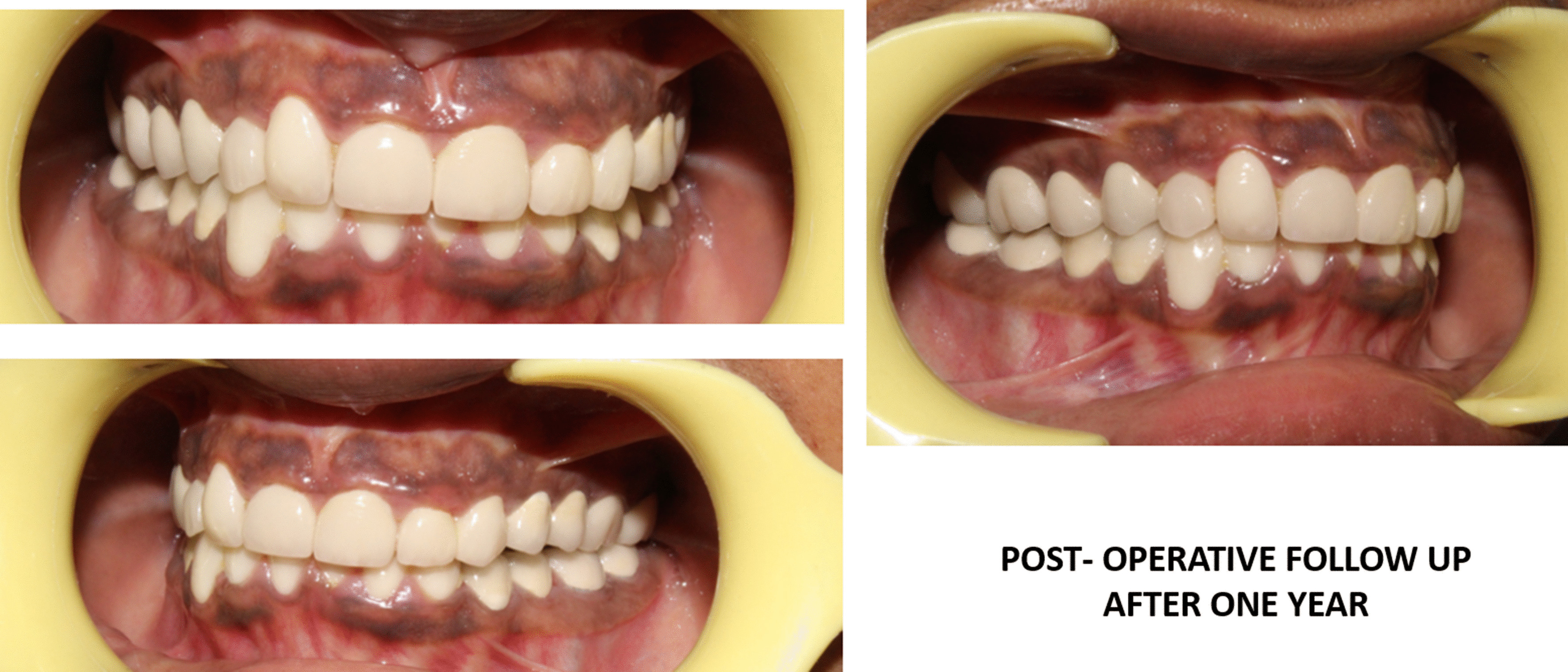
Postoperative follow-up clinical photographs after 1 year

**Fig. 5.**
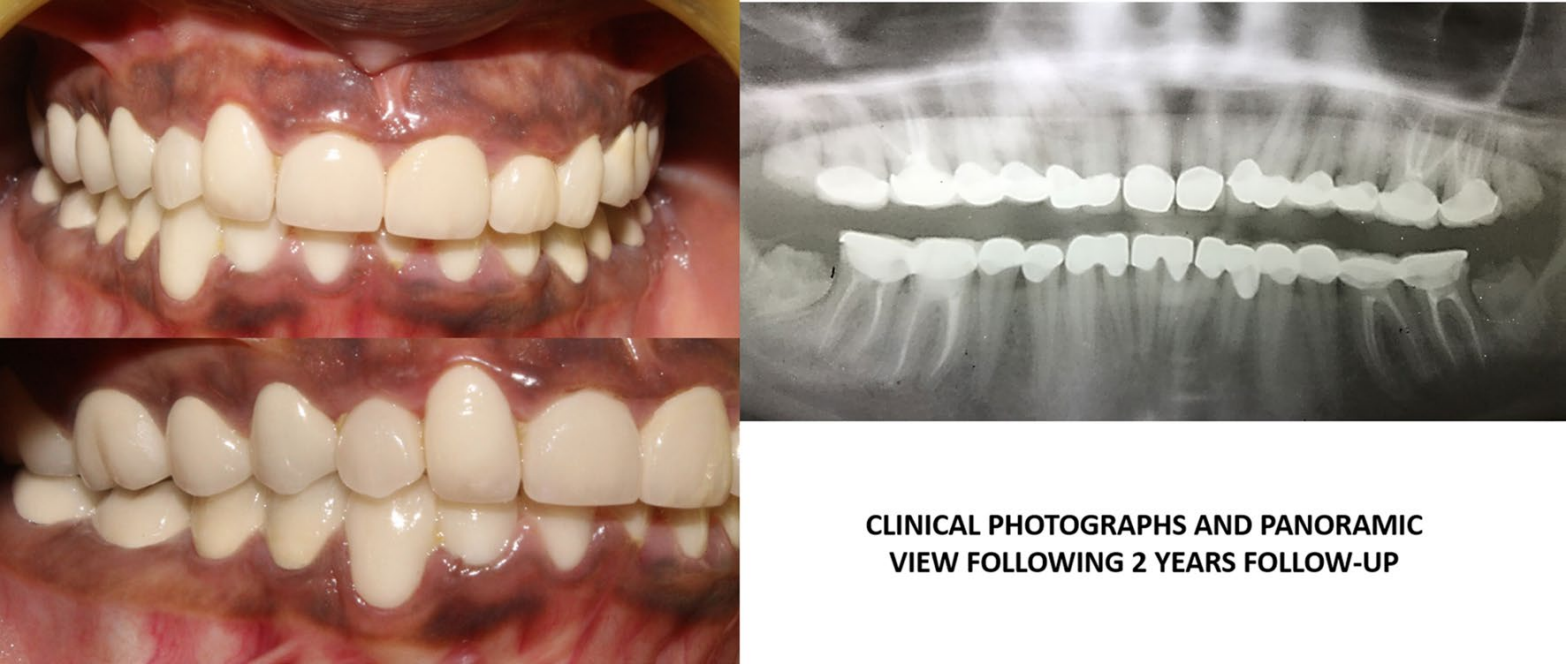
Postoperative follow-up clinical photographs and orthopantomogram view after 2 years

*Prosthetic treatment* included the tooth preparation on #17, 16, 15, 14, 24, 25, 26, 27, 34, 35, 36, 37, and 44, 45, 46, 47 to receive metal ceramic crowns. Diagnostic impressions were made using alginate (3M ESPE Alginate Impression Material). The diagnostic casts were then mounted on semi adjustable articulator (Hanau Articulator, Teledyne Hanau Buffalo, NY, USA) using Hanau facebow. The tooth preparations were performed quadrant wise so that the patient was adjusted with the new occlusion. First the posterior teeth were prepared and the temporary acrylic bridges (Integrity™ Temporary Crown & Bridge Material, DENTSPLY, USA) were fixed to increase the occlusal height. Temporary acrylic crowns were placed for 1 month to assess the patient’s forbearance to the new occlusion. Once the patient was habituated to the new occlusal height, then final maxillary and mandibular impressions were prepared with poly vinyl polysiloxane impression material (Aquasil Ultra Putty Soft Regular and Aquasil Ultra LV, Dentsply USA). Interocclusal records in centric occlusion were taken and the working casts were mounted on semi-adjustable articulator (Hanau Articulator, Teledyne Hanau Buffalo, NY, USA). Trial with metal ceramic restorations was done and finally cemented with self-adhesive resin (RelyX Unicem 2 Clicker A2 Universal Self-Adhesive Universal Resin, 3M-ESPE, USA). Then the anterior teeth were prepared for metal ceramic restorations. The patient was regularly monitored at 1 week, 1 month, 3 months, 6 months and at 1 year and 2 years intervals using clinical and radiographic examinations (Figs. 3, 4 and 5). Patient was highly satisfied with the treatment outcome and the gingival condition was maintained, esthetic impairment and the dentinal sensitivity was resolved.

## Discussion and review of literature

This case scenario presents with hypoplastic type of amelogenesis imperfecta which is completely due to genetic mutation and not hereditary. Amelogenesis imperfecta can be isolated or accompanied with other syndromes. It can be due to single gene mutation or various chromosomal aberrations. Such case reports are rare and not reported on regular basis.

Amelogenesis imperfecta (AI) is a herd of genetic disorders affecting the quantitative and qualitative aspects of the enamel formation [6]. The effects of amelogenesis imperfecta can be observed in both permanent and deciduous dentition. The mildest form of AI presents with discoloration, whereas in the most drastic form, it shows pitting, and appears to be hypomineralized. Enamel defects can be highly vacillating. It can range from defective enamel formation to complete deficiency in mineral and protein content [7].

AI is considered to be a category of genetic susceptible diseases involving defective genesis and maturation of enamel. The presence of defective enamel without any underlying systemic defects is the characteristic trait of AI. AI is the type of heritable abnormality which completely affects the ectodermal component, while the mesodermal component of the teeth remains normal. This type of abnormality can be either autosomal dominant, autosomal recessive or X-linked mode of inheritance [8]. The highly acceptable classification for AI was presented by Witkop in 1988, which was later modified by Nusier in 2004 [9]. AI is systematized into 4 patterns: hypoplastic, hypomaturation, hypocalcified, and hypomaturation-hypoplastic. Clinical signs and symptoms noticed are dentinal sensitivity, loss of vertical dimension, and esthetics. Treatment planning in AI involves a comprehensive diagnosis and treatment using an interdisciplinary approach of periodontal, prosthodontic and restorative treatment.

The entire treatment plan for a patient suffering from amelogenesis imperfecta (AI) should be aimed at protecting the entire stomatognathic system and restoration of hard tissues. The final treatment modality till date still revolves around either full coverage restorations or, more of adhesive restorations [10].

Amelogenesis imperfecta (AI) constitutes a collection of hereditary disorders where genetic mutations takes place in amelogenin, enamelin, and kallikrein-4 genes [11]. Further mutational investigation of individuals with AI, detailed comprehensive taxonomy system can be procured for this syndrome that will involve molecular depiction as well as a mode of inheritance and phenotype [12]. At the clinical interface, AI can be further divided into various forms based on disturbance in enamel formation as hypoplastic, hypo mineralized or hypo maturation type [13].

In some instances, in X-linked form, the defect is a consequence of genetic mutation in the amelogenin gene, AMELX. In dominant cases of AI, the enamelin gene, ENAM, is involved in the pathologic process [14].

In the hypomaturation type, the affected teeth presents with mottling, opaque white-brownish yellow discoloration, and is highly brittle. The radiographic picture shows reduced enamel thickness, but the density of enamel is identical to dentin [9].

The hypocalcified type manifests pigmented, softened, and loosened enamel. Radiographic analysis of this defect shows normal enamel thickness, but reduced density which is less than that of the dentin. In the hypoplastic type, the normal enamel maturation but thickness is reduced [15]. Further analysis of the AI's patterns is desirable especially the hypoplastic one which concerns our case report.

In our case, tooth impaction, delayed eruption, shortened roots and brownish discolored dentin were present. According to various case reports, around 80% of the patients with AI have the presence of impacted teeth [16–18]. Seow in his various observations, stated that people with amelogenesis imperfecta are more frequently associated with impacted permanent teeth [18].

Several authors [19–22] in their work suggested the need for porcelain fused to metal restorations as the treatment modality for their patients. With advancing technology, the full ceramic restorations are also preferred [23, 24]. With the increasing need for better esthetics and ability of bonding to dentin, porcelain fused to metal crowns has been well acceptable by the practitioners to restore the form and function to a satisfactory level [19, 25]. Providing laminate veneers to such patients can be disadvantageous. The marginal adaptation, and problems in bonding would give rise to further periodontal problems with laminate veneers [26, 27]. In this present clinical scenario, full-mouth porcelain fused to metal restorations were preferred for enhanced stability, mechanical durability, satisfactory esthetics and protection of the remaining dentin. Due to the lack of horizontal overlap, laminate veneers was not considered as a viable option.

Restoring the smile in individuals with AI deals with lot of accuracy, patience and skill along with comprehensive collaboration with various sectors of dental treatment. Multidisciplinary approach of treatment planning includes restoration of function, aesthetics and vertical dimension.

During treatment planning several various factors have to be taken into consideration, such as age of the patient, oral hygiene status, quality of life, periodontal considerations, internal anatomy of teeth, remaining tooth structure, and orthodontic consideration [27, 28]. Taking the different factors into account, the treatment modalities may vary. The task of performing root canal treatment in patients with AI is considered to be difficult due to the anatomy of teeth. Pulp space therapies along with restorative treatment modalities should always be considered. Although, the teeth affected in AI mostly have radiographic obliteration, the teeth appear to be normal and the pulp does not become nonvital because of excessive production of dentin. In most instances, endodontic therapy is considered mandatory in patients with AI. Elective endodontic treatment in such patients may produce long term success rates and improve the prognosis of the treatment. With respect to this case report, the endodontic treatment was not difficult, though there was presence of MB2 in maxillary molars, and calcification. But with the correct infection control protocol and irrigation sequence, and profound use of EDTA, pulp space therapies were successfully performed. Rotary instrumentation provided better canal visibility and corresponding single cone obturation added to the success of the treatment and prevented the chances of perforation. Full coverage post endodontic restorations are best preferred to such patients because they restore and protect the dental tissues from further destruction.

## Conclusion

Complete reintegration of the patient with amelogenesis imperfecta is a constant challenge to the clinician which requires utmost care, precision and interdisciplinary approach with active participation of various branches of dentistry. The multidisciplinary approach of these conditions should be oriented towards maintaining the functional, esthetic and physical wellbeing of the patient.

## Data Availability

Not applicable.

## Abbreviations

AI: Amelogenesis imperfecta
OPG: Orthopantomogram
IOPA: Intraoral periapical radiograph
MB2: Mesiobuccal 2
AMELX gene: Amelogenin gene
ENAM gene: Enamelin gene
EDTA: Ethylenediaminetetraacetic acid

## References

[1] Robinson C, Briggs HD, Atkinson PJ, Weatherell JA. Matrix and mineral changes in developing enamel. J Dent Res. 1979;58:871–82. 10.1177/00220345790580024101 283129

[2] Simmer JP, Fincham AG. Molecular mechanisms of dental enamel formation. Crit Rev Oral Biol Med. 1995;6:84–108. 10.1177/10454411950060020701 7548623

[3] Mahoney EK, Rohanizadeh R, Smail FSM, Kilpatrick NM, Swain MV. Mechanical properties and microstructure of hypomineralized enamel of permanent teeth. Biomaterials. 2003;25:5091–100. 10.1016/j.biomaterials.2004.02.044 15109872

[4] Chaudhary M, Dixit S, Singh A, Kunte S. Amelogenesis imperfecta: report of a case and review of literature. J Oral Maxillofac Pathol. 2009;13(2):70–7. 10.4103/0973-029X.57673 PMC3162864 21887005

[5] Paine ML, White SN, Luo W, Fong H, Sarikaya M, Snead ML. Regulated expression dictates enamel structure and tooth function (review). Matrix Biol. 2001;20:273–92. 10.1016/s0945-053x(01)00153-6 11566262

[6] Witkop CJ. Amelogenesis imperfecta, dentinogenesis imperfecta and dentin dysplasia revisited: problems in classification. J Oral Pathol. 1988;17(9–10):547–53. 10.1111/j.1600-0714.1988.tb01332.x 3150442

[7] Chanmougananda SC, Ashokan KA, Ashokan SC, Bojan AB, Ganesh RM. Literature review of amelogenesis imperfecta with case report. J Indian Acad Oral Med Radiol. 2012;24:83–7.

[8] Chaudary M, Dixit S, Singh A, Kunte S. “Amelogenesis imperfecta”—report of a case and review of literature. J Oral Maxillofac Pathol. 2009;13(2):70–7. 10.4103/0973-029X.57673 PMC3162864 21887005

[9] Canger EM, Celenk P, Yenisey M, Odyakmaz SZ. Amelogenesis Imperfecta, hypopalstic type associated with some dental abnormalities: a case report. Braz Dent J. 2010;21(2):170–4. 10.1590/s0103-64402010000200014 20640366

[10] Roma M, Hegde S. Amelogenesis imperfecta: a review of the literature. J Pharm Sci Res. 2016;8(9):1042–4.

[11] Patel M, McDonnell ST, Iram S, Chan MFW-Y. Amelogeneis Imperfecta-lifelong management. Restorative management of the adult patient. Br Dent J. 2013;215(9):449–57. 10.1038/sj.bdj.2013.1045 24201615

[12] Crawford PJM, Aldred M, Bloch-Zupan A. Amelogenesis imperfecta. Orphanet J Rare Dis. 2007;2(17):1–11. 10.1186/1750-1172-2-17 PMC1853073 17408482

[13] dos Santos MCLG, Line SRP. The genetics of amelogenesis imperfecta: a review of the literature. J Appl Oral Sci. 2005;13(3):212–7. 10.1590/s1678-77572005000300002 20878018

[14] Nigam P, Singh VP, Prasad KD, Tak J. Amelogenesis imperfecta: a case report and review of literature. Int J Sci Stud. 2015;2(10):146–9.

[15] Patil PG, Patil SP. Amelogenesis imperfecta with multiple impacted teeth and skeletal class III malocclusion: complete mouth rehabilitation of a young adult. J Prosthet Dent. 2014;111:11–5. 10.1016/j.prosdent.2013.06.010 24210728

[16] Reddy SS, Aarthi Nisha V, Harish BN. J Clin Exp Dent. 2010;2(4):e207–11.

[17] Helen Y, Patel J, Kwok J. Amelogenesis imperfecta, hypodontia and impacted teeth, a challenging case for rehabilitation. Clin Oral Impl Res. 2017;28(Suppl. 14).

[18] Seow WK. Dental development in amelogenesis imperfecta: a controlled study. Pediatr Dent. 1995;17:26–30. 7899098

[19] Gökçe K, Canpolat C, Özel E. Restoring function and esthetics in a patient with amelogenesis imperfecta: a case report. J Contemp Dent Pract. 2007;8:90–101. 17486193

[20] Toksavul S, Ulusoy M, Türkün M, Kümbüloğlu Ö. Amelogenesis imperfecta: the multidisciplinary approach: a case report. Quintessence Int. 2004;35:11–4. 14765635

[21] Turagam N, Mudrakola DP. Restoring esthetics and function in a patient with amelogenesis imperfecta—a multidisciplinary approach. Dentistry. 2015;5:3.

[22] Sadighpour L, Geraminapah F, Nikzad S. Fixed rehabilitation of an ACP PDI class III patient with amelogenesis imperfecta. J Prosthodont. 2009;18:64–70. 10.1111/j.1532-849X.2008.00368.x 18798781

[23] Kostoulas I, Kourtis S, Andritsakis D, Doukoudakis A. Functional and esthetic rehabilitation in amelogenesis imperfecta: a case report. Quintessence Int. 2005;36:329–38. 15892529

[24] Christensen GJ. Porcelain-fused-to-metal versus nonmetal crowns. J Am Dent Assoc. 1999;130:409–11. 10.14219/jada.archive.1999.0211 10085664

[25] Ozturk N, Sari Z, Ozturk B. An interdisciplinary approach for restoring function and esthetics in a patient with amelogenesis imperfecta and malocclusion: a clinical report. J Prosthet Dent. 2004;92:112–5. 10.1016/S0022391304002288 15295316

[26] Sari T, Usumez A. Restoring function and esthetics in a patient with amelogenesis imperfecta: a clinical report. J Prosthet Dent. 2003a;90:522–5. 10.1016/j.prosdent.2003.08.024 14668751

[27] Sari T, Usumez A. Restoring function and esthetics in a patient with amelogenesis imperfecta: a clinical report. J Prosthet Dent. 2003b;90(6):522–5. 10.1016/j.prosdent.2003.08.024 14668751

[28] Shetty N, Joesph M, Basnet P, Dixit S. An integrated treatment approach: a case report for dentinogenesis imperfect Type II. Kathmandu Univ Med J. 2007;5(2):230–3. 18604025

